# Simulation test on water and salt transport characteristics of saline soil in Uzbekistan under large temperature difference

**DOI:** 10.1371/journal.pone.0336386

**Published:** 2025-11-14

**Authors:** Fang Zheng, Wenqiang Li, Zhanping Song, Dongdong Xu, Junbao Wang, Yan Wang, Yuwei Zhang

**Affiliations:** 1 School of Civil Engineering, Xian University of Architecture and Technology, Xian, China; 2 Shaanxi Key Laboratory of Geotechnical and Underground Space Engineering, Xian, China; University of Sharjah, UNITED ARAB EMIRATES

## Abstract

To investigate the characteristics of water and salt migration (WSM) in saline soils subjected to climate conditions characterized by large temperature differences. In this paper, salted soil highway roadbeds in Uzbekistan were selected as the background for the study, and the salted soils in the region of the site were remodeled through a field study and based on field data. Subsequently, utilizing the assembled laboratory test apparatus, a model test of the WSM on the remodeled saline soil was carried out to analyze the behavior of the WSM in response to large temperature differences, the resulting soil deformation, and the influence of varying cold-end temperatures and alternating cooling and heating times on the WSM. The findings indicated that the temperature profile with in the soil column (SC) exhibits a “wavy” pattern as a result of thermal conductivity. During the freezing phase, water migrates from the lower section to the upper section in response to the temperature gradient and soil-water potential. Conversely, during the melting phase, water diffuses and migrates from the upper section downward in the form of steam molecules due to the action of evaporation. This process culminates in a “C” shape distribution of the curve of the water content inside the SC. During the freezing process, salt migrates from the bottom to the top along with the liquid water. Conversely, during the melting process, salt accumulates at the top of the SC accumulates. This phenomenon results in an “S” distribution of the salt content inside the SC. At the end of the freeze-thaw cycle (FTC), the deformation of the saline soil increased with the decrease of the cold end temperature, and the alternating time between heat and cold did not produce significant changes in the deformation of the saline soil. The moisture content at the upper end of the SC increases with decreasing cold-end temperature. The increase in the number of FTCs exacerbates the salinization of the soil in a certain part of the interior of the SC. The findings of this research may offer a theoretical foundation and technical assistance for the design of subgrade structures in saline soils subjected to climates characterized by large temperature differences.

## 1. Introduction

Saline soil, a type of geological material that is particularly sensitive to temperature changes, is widely distributed worldwide and is found in more than 100 countries [1]. Owing to large temperature differences, especially in regions characterized by a highly cold and dry climate, the effects of FTCs cycles and evaporation are significant, leading to the ongoing movement and accumulation of soil salts at the surface. In this process, the presence of salt adversely affects the construction of the project [2–4]. On this basis, most scholars have researched the influence of the temperature field on projects in seasonally frozen areas and have achieved remarkable results [5,6].

Researching the forces driving WSM, Zhang et al. summarized four hypotheses of the forces driving water migration in saline soil and three main modes of migration of salt ions from the literature [7]. The hypotheses of the forces driving water migration are as follows: the ① capillary action hypothesis; ② pellicular water migration theory; ③ force of crystallization theory; and ④ adsorption-film theory. The modes of salt migration are as follows: ① seepage migration; ② dispersion and migration; and ③ seepage–dispersion migration. Everett utilized capillary theory to elucidate the process of water transportation during the process of soil freezing [8]. Harlan suggested that the soil water potential gradient is the driving force of water migration in frozen soil. On the basis of the principle of hydrodynamics, he proposed a coupled hydrothermal mathematical model to describe water transportation during soil freezing [9]. Taber et al. proposed crystallization force theory and noted that during the process of ice crystallization, a pressure gradient is generated in the ice–water system, which causes the liquid water to migrate in the direction of ice crystal growth [10,11]. On the basis of an experiment, Bresler reported that diffusion caused by convection and a concentration gradient can occur simultaneously in the process of solute migration, and the direction of migration may be the opposite or the same [12]. Beskow proposed the adsorption-film theory, which posits that water migration in frozen soil is carried out along the unfrozen film of water between soil particles and ice [13]. Wu et al. studied the laws of WSM and characteristics of the deformation of salinized frozen soil and reported that water is the medium for salt migration, that salt dissolved in unfrozen water is prone to percolation and migration, that WSM upward during the cooling period and reverse during the warming period, and that temperature, water, salt and soil deformation are coupled [14]. Hird concluded through experiments that salt diffusion was the cause of soil salinization [15].

In research on the WSM under freezing conditions, Xu et al. discussed the effects of the freezing point and temperature gradient on the salt distribution via one-way freezing tests [16]. Cary et al. analyzed the patterns of migration of different salt ions in the frozen state, combined with laboratory tests, and the results revealed that a temperature gradient would induce the migration of salt to the cold end of the soil [17]. Xu et al. studied the WSM in frozen soil through laboratory experiments and reported that both water and salt migrated from bottom to top during the freezing process [18]. Bing et al., on the basis of a one-way freezing test of saline soil, reported that during the freezing process, an isolated freezing front forms inside the saline soil, preventing salt from migrating to the cold end of the soil [19]. Baker et al. reported that the repellant salt content at the freezing interface decreased as the freezing rate decreased [20]. Li et al., from an experimental study of water migration under freezing conditions, noted that when there is an external water supply in a soil sample, liquid water with a low temperature preferentially condenses into ice, and the water in the lower soil migrates upward to supplement it, resulting in a higher water content at the top than the initial water content [21]. Morgenstern conducted experiments on water migration in frozen soil under closed and open systems, and the controlled temperature gradients were different during the experiments. The results revealed that the amount of water that migrated gradually increased with increasing temperature gradient [22]. Bing et al. studied the redistribution of salt during FTCs and reported that the mechanism of water movement largely determines the salt migration mechanism [23,24].

With reference to the WSM under evaporative conditions, Chen et al. discussed the effects of evaporation and capillary action on the internal pore structure of saline soil based on mercury intrusion tests [25]. Yang et al. studied the pattern of WSM in saline soil under evaporative conditions via laboratory tests. The findings indicate that as the temperature rises, the transport rates of water and salt increase to different degrees under the double excitation of capillary action and the temperature gradient, and salt mainly accumulates in the middle and upper layers of the SC [26]. Chen et al. observed that the process of evaporation results in the migration of a portion of the water present in the upper soil into the atmosphere, and the other part of the water migrates downward with salt, resulting in the accumulation of salt at the base of the soil [27]. On the basis of laboratory tests, Zhang et al. noted that when the temperature increases, water at the bottom of the soil migrates upward under the action of evaporation, whereas salt migrates to the top of the soil, with water as the carrier, resulting in salinization of the upper soil [28]. Zhou et al. observed that temperature exerts a significant influence on the solubility of salt, subsequently impacting its dissolution and adsorption processes. This, in turn, results in the redistribution of salt within the soil matrix. It was further noted that as the salt nears the high-temperature end of the temperature range, its distribution increases concomitantly. The dispersion and suction of water in the pores by matric suction facilitate the continuous migration of water toward the colder end of the temperature spectrum [29].

The above research shows that in the freezing stage, WSM is driven mainly by the temperature gradient, and water carries salt to the cold end. In the melting stage, WSM are affected by evaporation, and accumulation occurs at different depths in the soil, whereas WSM under large temperature differences can be regarded as the processes of freezing at low temperatures and evaporation at high temperatures. The water and salt exhibit different migration patterns during freeze and melting, so severe engineering accidents often occur during the construction of engineering areas in saline soil in climates with large temperature differences. In view of this information, in this study, northwestern Uzbekistan was selected as the research area, the local saline soil was reshaped according to the onsite survey data, and modeling of WSM by means of an indoor WSM tester; in addition, the effects of different cold-end temperatures and alternating cooling and heating times on the WSM patterns under large temperature differences were obtained by indoor WSM tests.

## 2. Water-salt migration device design

**Fig 1:**
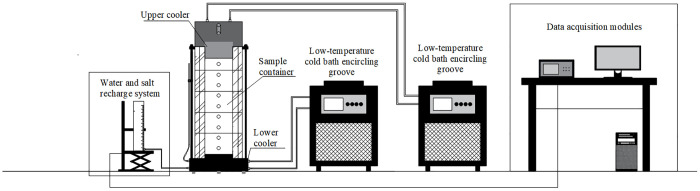
Schematic diagram of WSM test simulator.

To explore the pattern of WSM in sulfate-rich saline soil under extreme climate conditions, this paper assembled a WSM model test device based on the existing WSM test. (. 1). The device system is composed of the sample container, temperature simulation system, water and salt recharging device and data acquisition equipment. The experiment simulated the climatic conditions of freezing in winter and melting in summer via setting the temperatures of the FTC (Fig 1).

### 2.1. Sample container

Sample container design for the wall thickness of 20 mm plexiglass cylinder structure, test soil samples can be in the plexiglass cylinder in the layered compaction molding, and plexiglass cylinder body is uniformly set up with the sensor reserved holes. In the test of the plexiglass cylinder side wall of the sensor hole set up from the bottom of the plexiglass cylinder 3.5 cm, every 7.5 cm symmetrically set up a 5 mm diameter sensor orifice and the bottom * height of 38 mm * 13 mm sensor rectangular hole, a total of 6 orifice and 6 rectangular hole in the test reserved.

### 2.2. Temperature simulation system

The temperature simulation system mainly consists of two low-temperature cold bath circulating tanks, heat conduction tubes, upper refrigeration head, lower refrigeration head and circulating refrigeration liquid, whose function is to simulate the changes of the natural environment and provide the required test temperature for the SC specimen. Among them, the upper and lower refrigeration heads are designed as disk structure.

### 2.3. Device for water and salt supply

The device for recharging water and salt was composed of a Marriott bottle, a water guide hose and a weighing table, which provided the required water or salt environment for the SC in the test. During the test, the height of the water outlet on the Marriott bottle was adjusted by controlling the height of the weighing table to simulate different groundwater heights. To maintain a steady salt content, in this test, the liquid for replenishing the water and salt in the device was distilled water.

### 2.4. Data acquisition

**Fig 2:**
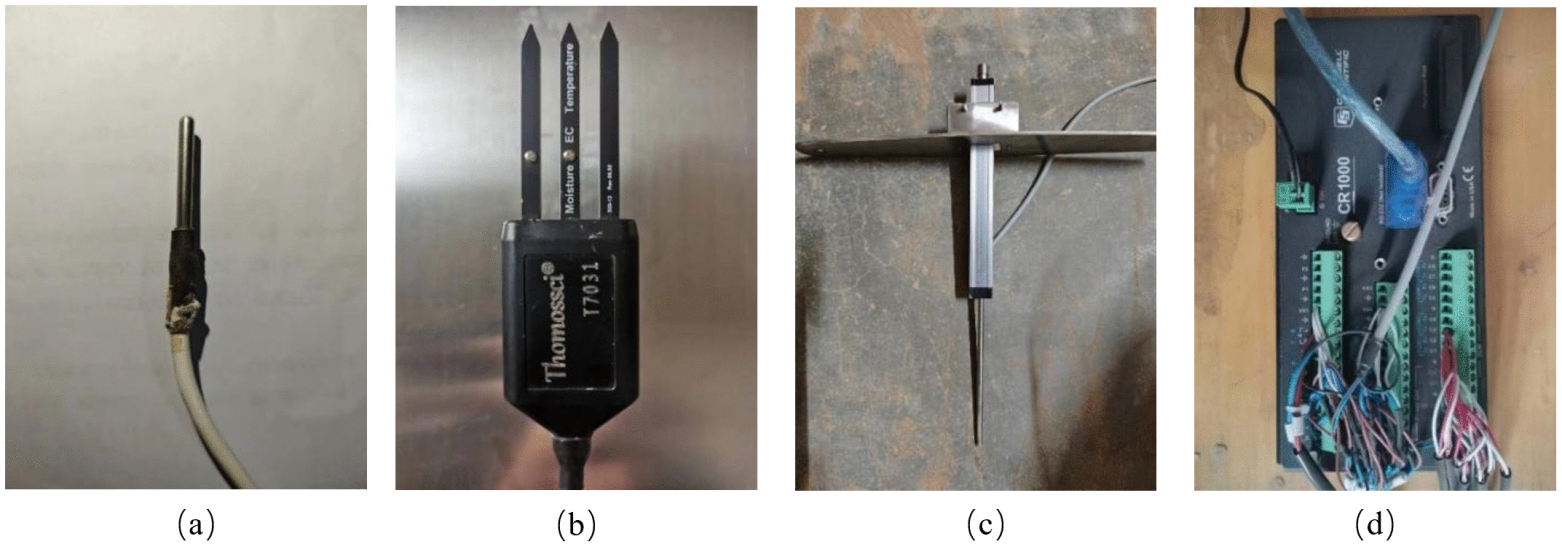
Data acquisition equipment: (a) Temperature sensor probe, (b) Three-in-one sensor probe, (c) Displacement transducer probe, (d) CR1000 data collector.

The data acquisition equipment in this test was composed of a temperature sensor, three-in-one sensor, displacement transducer and CR1000 data collector (as shown in Fig 2). The plexiglass cylinder side wall has 6 reserved sensor holes, after the SC is compacted, the probe of the 3-in-1 sensor is directly inserted into the SC, and the results detected by the sensor are used to indicate the internal temperature, water content and salt content of the SC during the test. The temperature sensor was used to monitor the temperature change inside the SC, the three-in-one sensor was used to monitor the change in the water content and salt content inside the SC, and the displacement sensor was used to monitor the deformation of the SC.

## 3. Basic properties of the material

### 3.1. Overview of the study area

**Fig 3:**
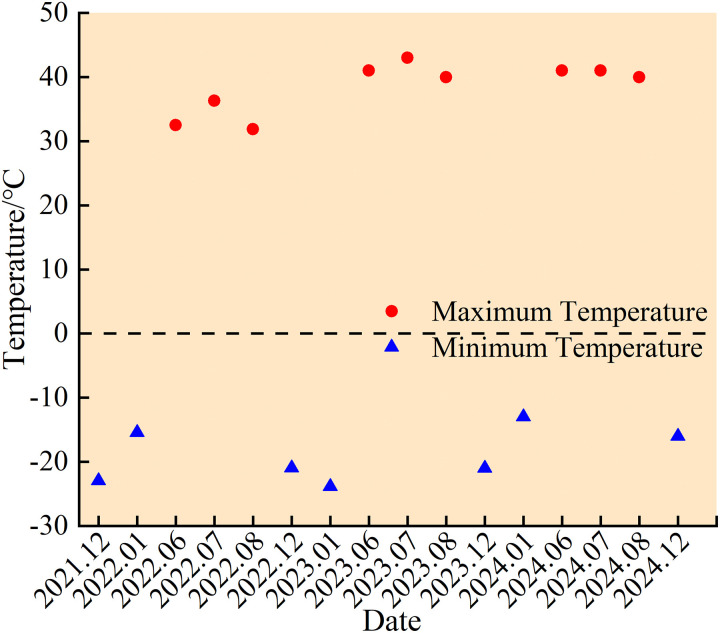
Temperature changes in the study area over the last three years.

This paper is based on the Salted Soil Highway Project in Uzbek, which is located in the northwestern part of the country, where not only the winters are cold and the summers are hot, but also a large amount of sulfate salty soil are distributed. The research area is located in the desert and Gobi region and has a continental climate with high temperature variation in the climate throughout the year and abundant wind energy. Fig 3 shows temperature data for the last three years in the northwestern part of the Uzbek, which shows that the area is hot in the summer, with maximum temperatures reaching 40°C. Winter is cold and prone to snowfall, and the lowest temperature of about −30°C. The average annual rainfall is approximately 100 mm.

### 3.2. Properties of regional soils

**Fig 4:**
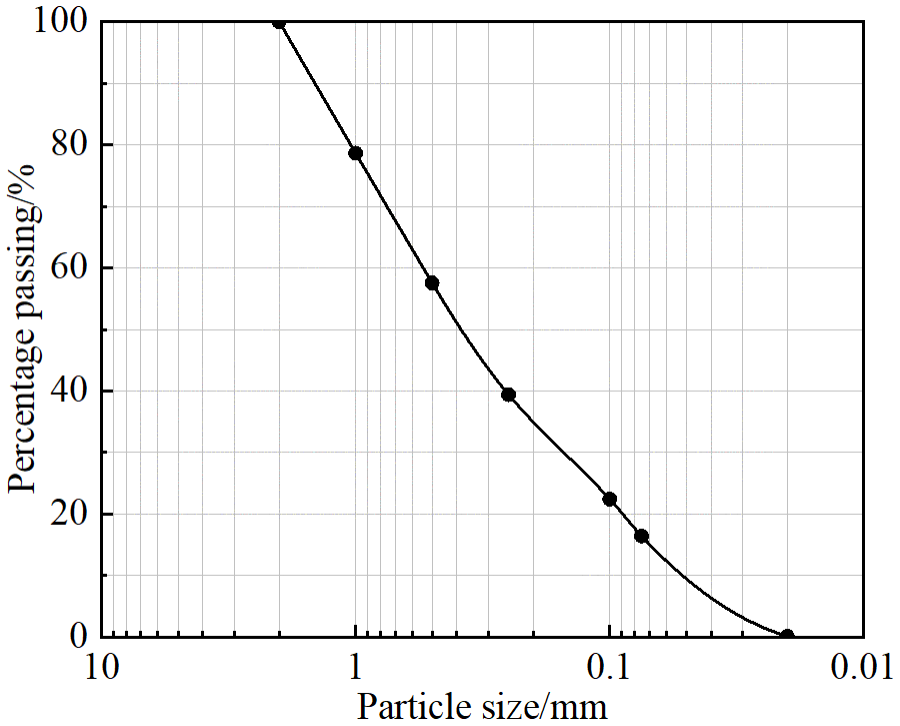
Particle size curve.

The basic physical properties of the soil in the study area were obtained via particle analysis tests, compaction tests and soluble salt tests. Fig 4 shows the grain size curve of the soil, and Table 1 shows the basic physical properties of the soil, in which the plastic limit of the soil is 19.61% and the liquid limit is 31.79%. In accordance with ASTM D2487 (2017), the soil is classified as clayey sand [30]. Table 2 shows the content of each ion in the soil. According to the relevant provisions in the Technical Specifications for Highway Subgrade Construction (JTG/T3610-2019), the ratio of chloride ions to sulfate ions in the soil sample is 0.239 < 0.3, and it is classified as sulfate saline soil according to its salt-bearing properties [31]. The initial salt content of the soil sample is 1.921%, which classifies it as a medium-salinity soil, according to the salt content.

**Table 1:** Basic physical properties of the soil samples.

Optimum water content(%)	Maximum dry density (g/cm^3^)	Liquid limit (%)	Plastic limit (%)	Plasticity index
17.56	1.79	31.79	19.61	12.18

**Table 2:** Contents of ions in the soil samples.

The content of each ion (%)							Cl^-^ (mmol/kg)	SO_4_^2-^ (mmol/kg)	Cl^-^/SO_4_^2-^
CO_3_^2-^	HCO_3_^-^	Cl^-^	SO_4_^2-^	Ca^2+^	Mg^2+^	K^+^+Na^+^			
0.000	0.061	0.106	1.200	0.291	0.079	0.184	2.986	12.492	0.239

### 3.3. Soil properties of remodeled soils

**Fig 5:**
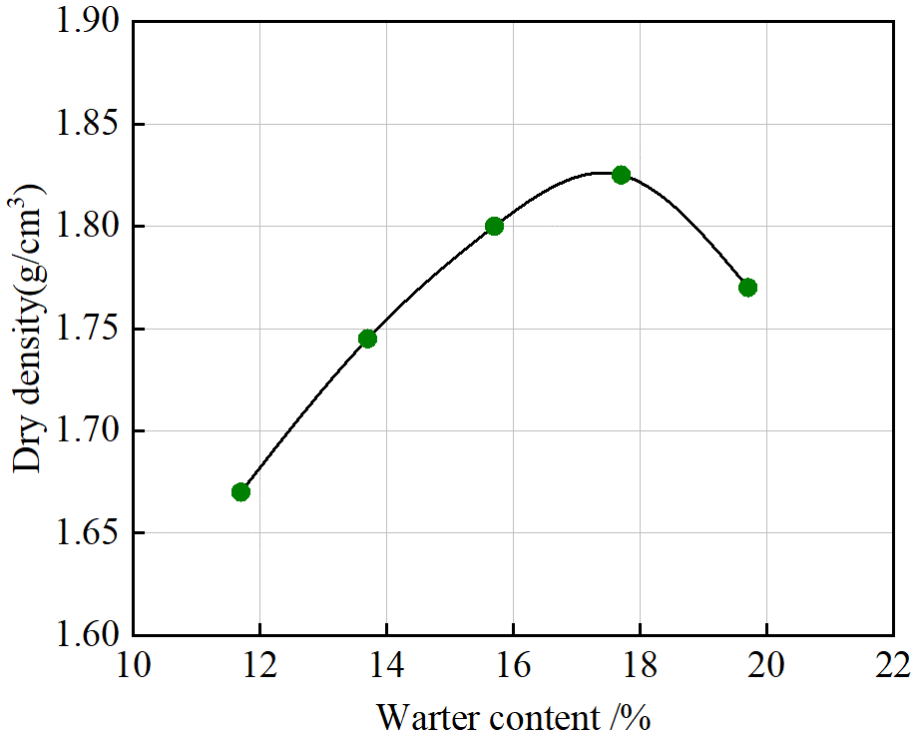
Compaction curve.

The soil used in this experiment was artificial saline soil that was prepared in the laboratory on the basis of the properties of the soil at the site in the study area. To ensure that the properties of the artificial saline soil correlate with the field soil, according to the grain size curve shown in Fig 4, the standard sand and loess were mixed at 3:7 and reconstructed into the saline soil sample used in the test in the laboratory. The standard sand used in the test was from the Chinese ISO standard sand produced by Xiamen Asio Standard Sand Company, and the loess was from the Q3 loess in the pit of a construction site in Xi’an. The basic physical properties of the remolded saline soil were measured via basic soil tests. Table 3 shows the results of the liquid plastic limit of the remolded saline soil, and Fig 5 shows the compaction curve of the remolded saline soil. The maximum dry density of reshaped saline soil was 1.83 g/cm3, and the optimal water content was 17.73%. The properties of the remolded saline soil were very close to those of the soil at the project site, so artificial saline soil prepared indoors with a sand to soil ratio of 3:7 was used.

**Table 3:** Results of the liquid and plastic limits of the soil sample.

Sand and soil proportion	Plastic limit	Liquid limit	Plasticity index
3:7	18.1%	31.05%	12.95

### 3.4. Sample preparation

When preparing sulfate salty soil, first sieve the loess and sand according to particle size. The particle sizes of the loess are as follows: 1–2 mm, 0.5–1 mm, 0.25–0.5 mm, 0.1–0.25 mm, 0.075–0.1 mm, and less than 0.075 mm. Standard sand is sieved into the following particle sizes: 1–2 mm, 0.5–1 mm, 0.25–0.5 mm, and 0.1–0.25 mm. The soil particles and standard sand are then dried and stored for later use. According to the amount of soil required for the test, the grain size curve of the soil sample and the ratio of sand to soil, the content of each standard grain size group was weighed into the soil container and mixed evenly. Then, a mass of anhydrous sodium sulfate and distilled water was weighed, and distilled water and anhydrous sodium sulfate were mixed to achieve a certain concentration of sodium sulfate solution. Finally, the sodium sulfate solution was sprayed onto the measured sandy soil so that the salt content of the prepared sulfate soil was 1.9% and the initial moisture content was 17.73%. After the preparation of the sulfate soil, the soil was placed in a cool area at room temperature for 72 hours to ensure the uniformity of the soil sample.

**Fig 6:**
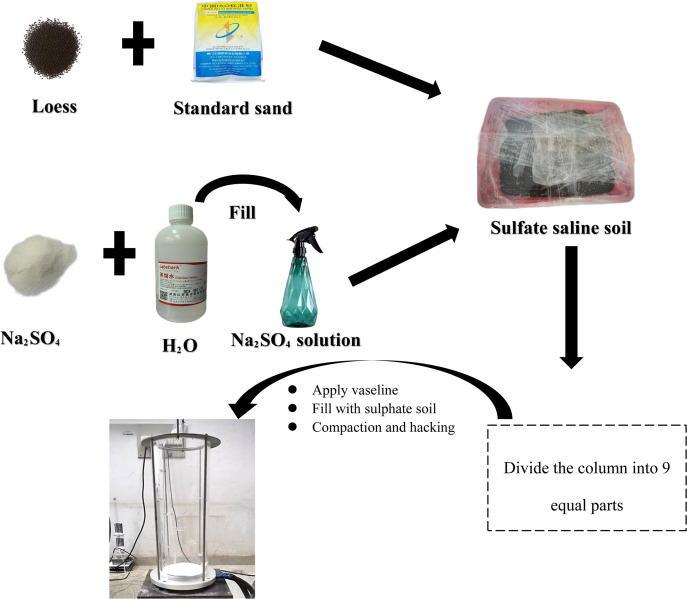
Flow chart of the sample preparation process.

When the soil sample was created, the mass of the sulfate soil used for the test was calculated first to ensure that the maximum dry density of the prepared soil sample was 1.83 g/cm^3^. Second, Vaseline was applied to the inner wall of the plexiglass cylinder to facilitate demolding after the test. Finally, the prepared sulfate soil was divided nine times into a sample cylinder, and each layer of the soil sample was compacted one way. After the compaction of each layer of the soil sample was completed, division was needed, and then the compaction of the next layer of the soil sample was carried out. The compaction degree of the soil sample was controlled at 94% in real time. A flow chart of the sample preparation is shown in Fig 6.

### 3.5. FTC test

In the test, the melting temperature at the top of the SC was set to 40°C, and the freezing temperatures at the top of the SC were set to −30°C, −25°C and −20°C. This FTC test consisted of 7 FTCs. Each time the SC experienced a 24-hour freezing stage and a 24-hour melting stage, a FTC was completed, and a total of 336h was required to complete a test. When the top temperature changed from −30°C, −25°C and −20°C to 40°C, the time required to set the temperature change (alternating cooling and heating times) was 2 hous. When the top temperature was changed from −30°C to 40°C, the alternating cooling and heating times were set to be 1 hour, 2 hours and 3 hours, respectively. The migration and deformation of water and salt in sulfate-rich soil under FTCs were studied by setting different temperature ranges and alternating cooling and heating times. There were five groups in this experiment, and the specific test scheme is shown in Table 4.

**Table 4:** Test scheme.

Test condition	Test number	Top freezing temperature	Top thawing temperature	Alternate cooling and heating	Salt content	Water content	Number of FTCs	Recharge source
Water and salt migration test	SC-A1	−30°C	40°C	2 h	1.9%	17.73%	7	Water
	SC-A2	−25°C						
	SC-A3	−20°C						
	SC-A4	−30°C		1 h				
	SC-A5			3 h				

### 3.6. Test process

**Fig 7:**
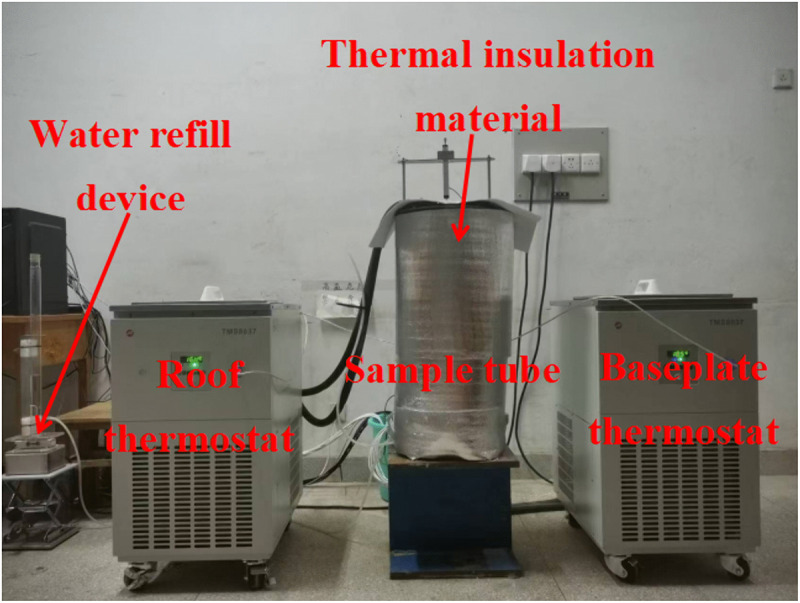
Test device for WSM.

The designed WSM device is shown in Fig 7. At room temperature, the artificially configured sulfate soil sample was loaded into the sample container. The upper and lower ends of the SCs were connected with a temperature simulation system, and the bottom end of the SCs was connected with a water and salt recharging device. The temperature at both ends of the SC in the test was controlled by two low-temperature cold bath circulation tanks, both of which were floor temperature-controlled and low-temperature constant-temperature devices produced by Zhejiang Tomos Technology Co., Ltd., model TMSB8037-R50. The temperature of the low-temperature cold bath circulation tank connected to the bottom of the SC was always maintained at 5°C, and an extreme climate environment was simulated by controlling the temperature of the low-temperature cold bath circulation tank connected to the top of the SC. In addition, insulation material was wrapped around the outer wall of the PVC sample pipe to prevent heat exchange between the soil sample and the external environment and reduce the effect of the temperature of the room on the test results.

## 4. Results and analysis

### 4.1. Change in the temperature distribution

**Fig 8:**
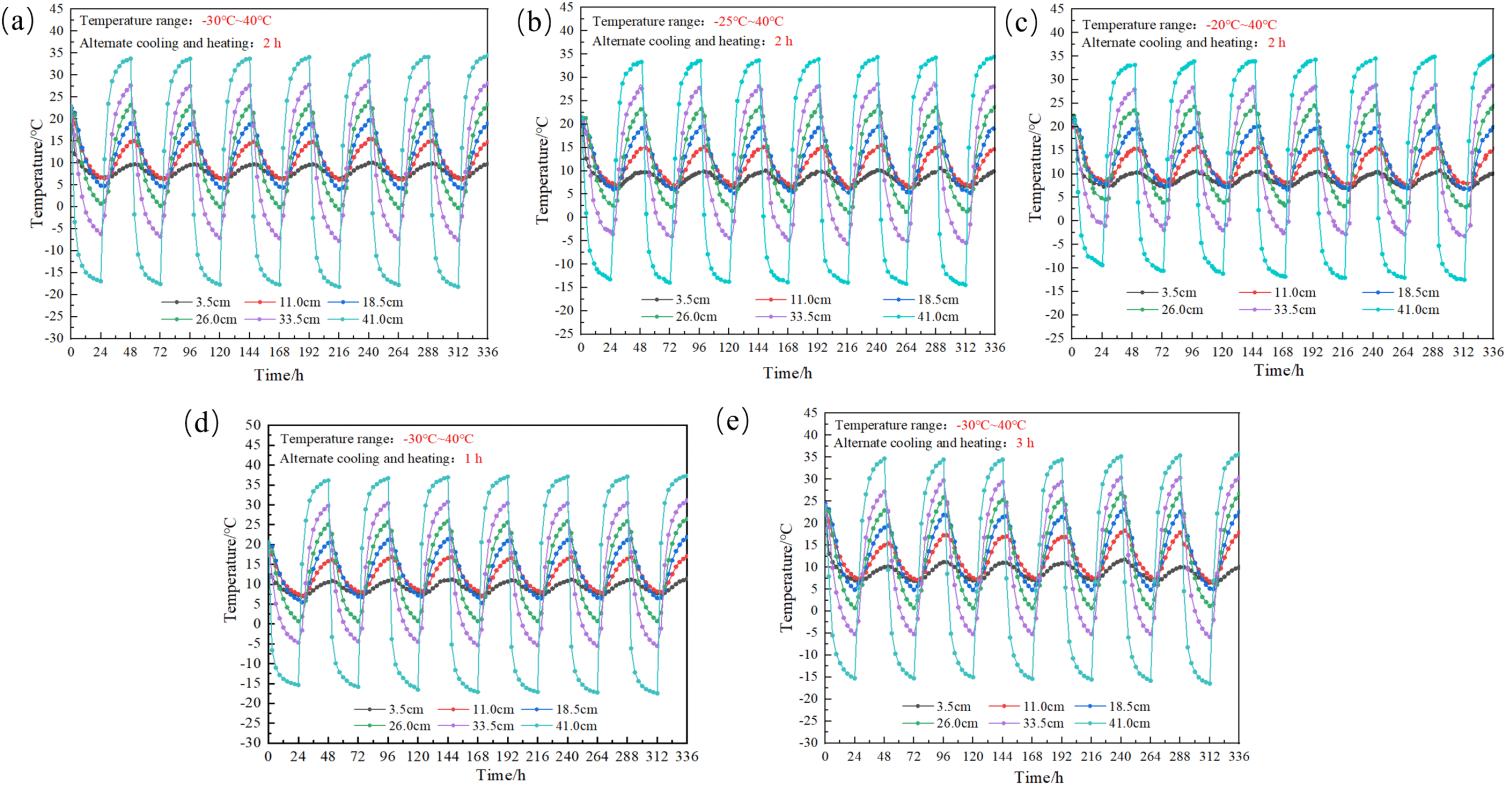
Temperature distribution in of the SC under conditions of large temperature differences: (a) SC-A1, (b) SC-A2, (c) SC-A3, (d) SC-A4, (e) SC-A5.

The temperature distribution inside the SCs A1-A5 under large temperature difference conditions is shown in Fig 8. In this test, the temperature at each measuring point inside the SC of the A1-A5 test group changes in the same way. The temperature distribution inside the SC is not uniform, and there is a temperature gradient between different measuring point heights. The temperature curves at each measuring point all show a “wavy” pattern over time, and the range of fluctuation in the temperature at each measuring point gradually decreases as the height of the measuring points in the SC decreases; that is, the greater the distance from the top of the SC is, the less influenced by the top temperature of the SC. According to Fourier’s theorem, the thermal conductivity is inversely proportional to the length of the thermal conduction path. As the length of the thermal conduction path increases, the heat transport gradually decreases. Therefore, the temperature decreases with increasing distance from the top of the SC.

Comparison of Fig 8(a)–(e) shows that at the end of the freezing stage, the temperature of each measurement point gradually decreases with time, and the closer the measurement point is to the top, the more obvious the temperature decrease is; at the end of the thawing stage, the temperature of each measurement point gradually increases with time, and the closer the measurement point is to the top, the more obvious the temperature increase is. Take Fig 8(a) as an example, at the end of the first freezing, the temperature at the 41.0 cm measuring point was −17.4°C, and at the end of the seventh freezing, the temperature at the 41.0 cm measuring point increased to −18.9°C; at the end of the first thawing, the temperature at the 41.0 cm measuring point was 34.2°C, and at the end of the seventh thawing, the temperature at the 41.0 cm measuring point decreased to 35.1°C. This is due to the fact that the FTC destroys the salinity of salt, and the temperature at each measuring point increases with time, and the closer the measuring point is to the top, the more obvious the temperature increase. This is because the FTC destroys the internal structure of saline-alkali soil, reducing the cohesion between soil particles and making the soil more loose. As the number of FTCs increases, the thermal conductivity of the soil gradually increases, ultimately leading to the above phenomenon.

### 4.2. Freezing front features

During the freezing process, the soil mass can be divided into a frozen zone, a frozen fringe zone and an unfrozen zone. The part between the frozen zone and the unfrozen zone is the frozen fringe zone [32], and the so-called freezing front is the frozen fringe zone. In the freezing stage, because the top of the SC is strongly affected by temperature, the temperature at the top of the SC preferentially decreases to the freezing point, resulting in the freezing front appearing earlier at the top of the SC than at the other parts. During the continuous freezing stage, other parts of the SC begin to reach the freezing point sequentially, resulting in the continuous downward movement of the freezing front inside the SC. When the freezing stage is over, the internal temperature of the SC decreases to the lowest temperature, and the downward movement of the freezing front reaches a maximum.

**Fig 9:**
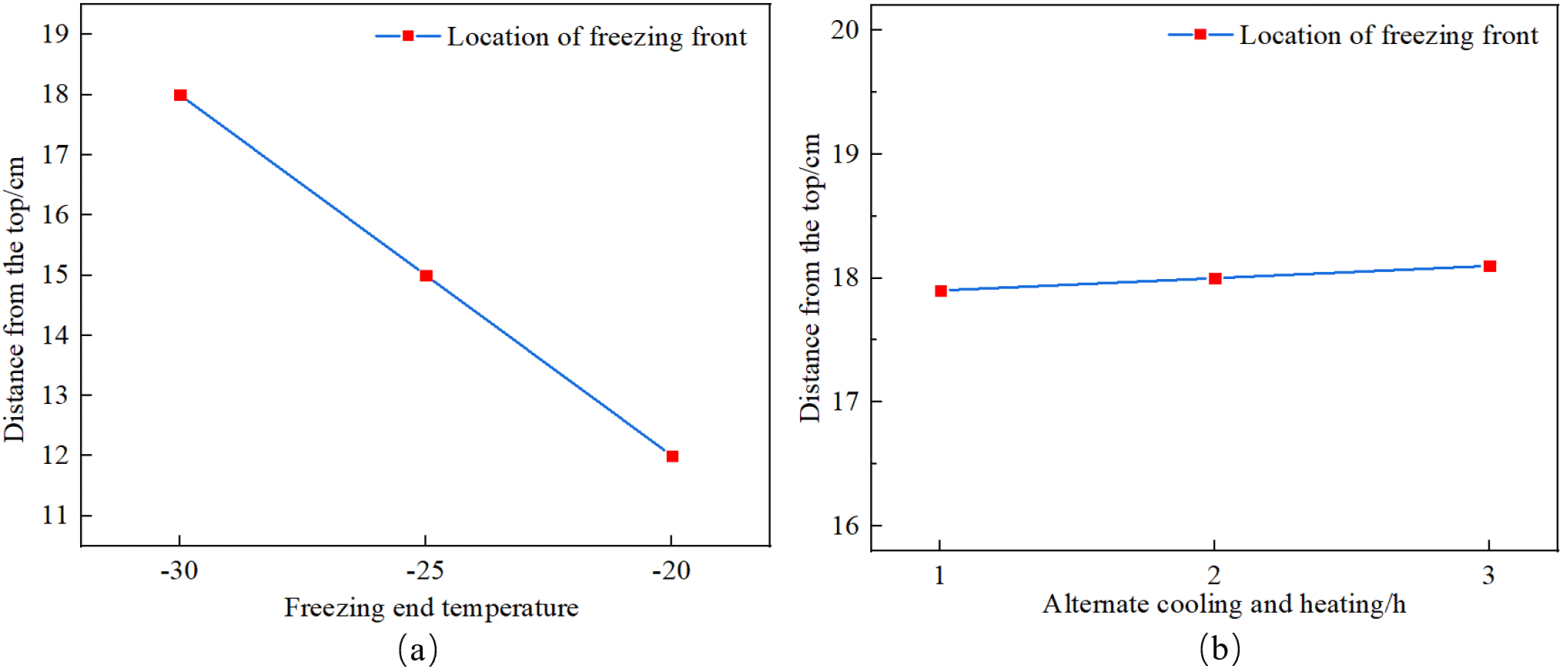
Graphical representation of the freezing front: (a)Location of freezing fronts at different temperature intervals, (b) Location of freezing fronts at different alternating cooling and heating times.

Fig 9 shows the location of the freezing front. As shown in Fig 9(a), for A1-3 SC samples with cold-end temperatures of −30°C, −25°C and −20°C, at the end of the freezing stage, the freezing front is located 18 cm, 15 cm and 12 cm away from the cold end, respectively, which indicates that as the temperature of the cold end decreases, the position of the freezing front moves further away from the cold end. This is because the lower temperature of the cold end intensifies the degree of freezing of the SC, resulting in an increasing depth of the freezing zone of the SC, and the position of the freezing front is also constantly moving downward. As shown in Fig 9(b), the greater the alternating cooling and heating times, the more the location of the freezing front is from the top, which indicates that the increase of alternating cooling and heating times causes the deepening of the freezing region of the SC.

### 4.3. Water distribution pattern

**Fig 10:**
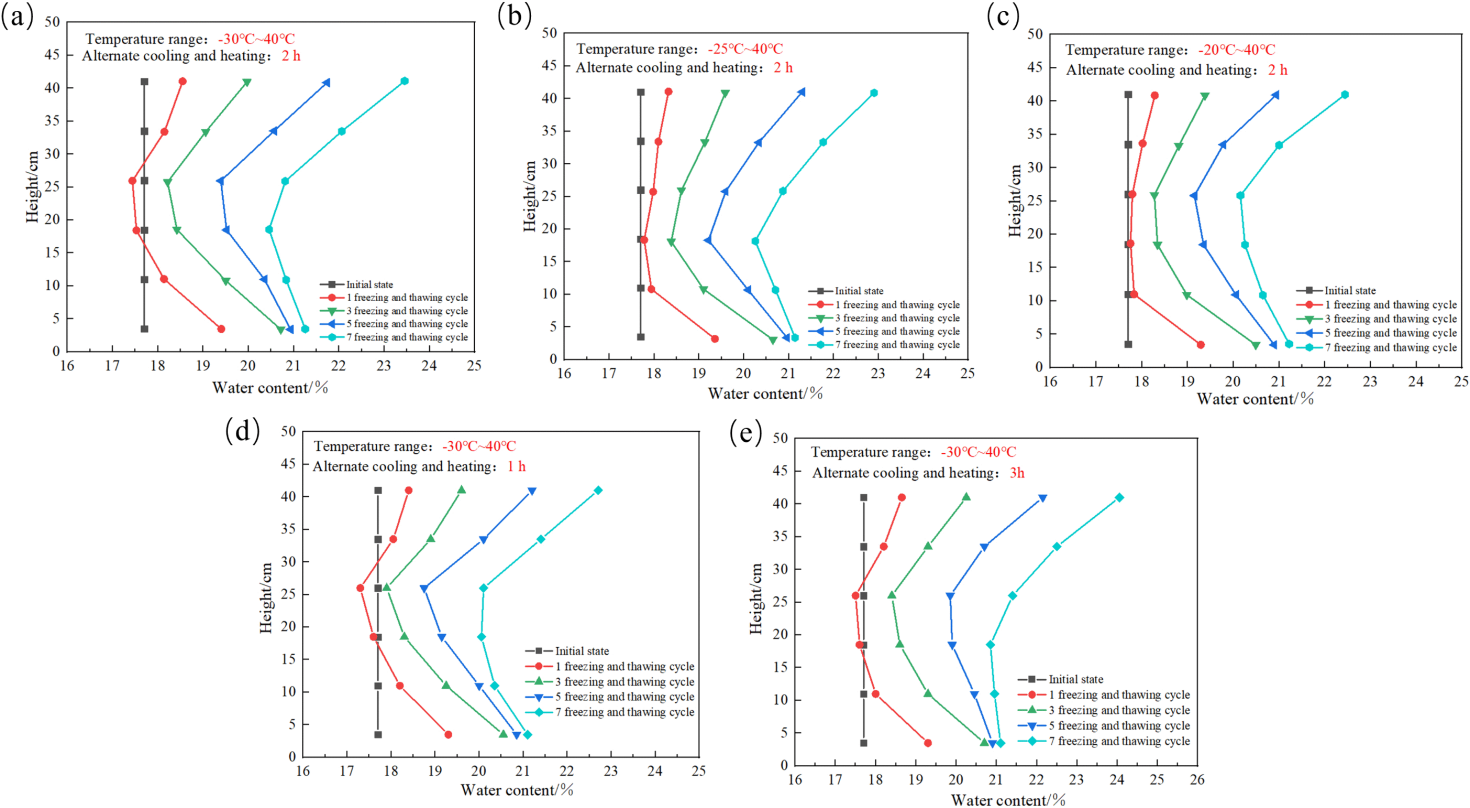
Moisture distribution within the soil column: (a) SC-A1, (b) SC-A2, (c) SC-A3, (d) SC-A4, (e) SC-A5.

The water distribution pattern inside the SC is shown in Fig 10. During 7 FTCs, the curve showing the water content distribution inside SC presents a “C”-type distribution; that is, the water contents at the bottom and top of the SC are greater than that in the middle of the SC. As the number of FTCs increases, the curve displaying the alteration in the water content of the SC advances rightward continuously, thereby indicating a continuous increase in the water content at each measuring point of the SC. As the number of FTCs increases to 3, the moisture content at the bottom of the SC gradually stabilizes, and when the number of FTCs increases to 5, the moisture content at the measuring point at 11 cm in the SC gradually stabilizes. This occurs because of the redistribution of water, salt and soil particles in the SC during the first freeze-thaw period, but no water‒salt migration channel forms in the SC, resulting in a large change in the water content at the bottom and top of the SC during the initial freeze-thaw period. With increasing number of FTCs, the pores between the soil particles increase in size, the soil gradually loosens, and the water storage capacity of the soil increases. The moisture content at each measurement point in the SC increases with increasing number of FTCs. When the number of FTCs reaches a certain number, the internal structure of the soil gradually stabilizes, and the WSM channels in the soil become gradually connected. Therefore, when the number of FTCs reaches 3, the water content at the 3.5 cm measuring point stabilizes, and when the number of FTCs reaches 5, the water content at the 11.0 cm measuring point stabilizes.

Comparison of Fig 10(a)–(c) shows that after 7 FTCs, the water content of the SC was lowest at 18.5 cm when the cold-end temperature was −30°C and −25°C, and lowest at 26.0 cm when the cold-end temperature was −20°C. It can be seen that the cold end temperature increases, the location of the lowest water content inside the SC also increases. When the cold end temperature increases from −30°C to −25°C, the water content at the top of the SC decreases from 23.6% to 23%; when the cold end temperature increases from −25°C to −20°C, the water content at the top of the SC decreases from 23% to 22.7%. It is interesting to note that the decrease in water content at the top of the SC is not the same when the temperature is also increased by 5°C. Based on the above results, it can be seen that the lower the temperature of the cold end, the stronger the water adsorption effect.

Comparing Fig 10 (a), (d) and (e), it can be seen that after 7 FTCs, the moisture content at the 41.0 cm measurement point was 22.7%, 23.6% and 24.1% when the alternating cooling and heating times was 1 hour, 2 hours and 3 hours, respectively. It can be seen that the longer the alternating cooling and heating times, the higher the moisture content at the top of the SC after seven FTCs. This is due to the fact that the unfrozen water inside the SC will have more time to migrate to the top of the SC when alternating cooling and heating times increases.

### 4.4. Pattern of salt distribution

**Fig 11:**
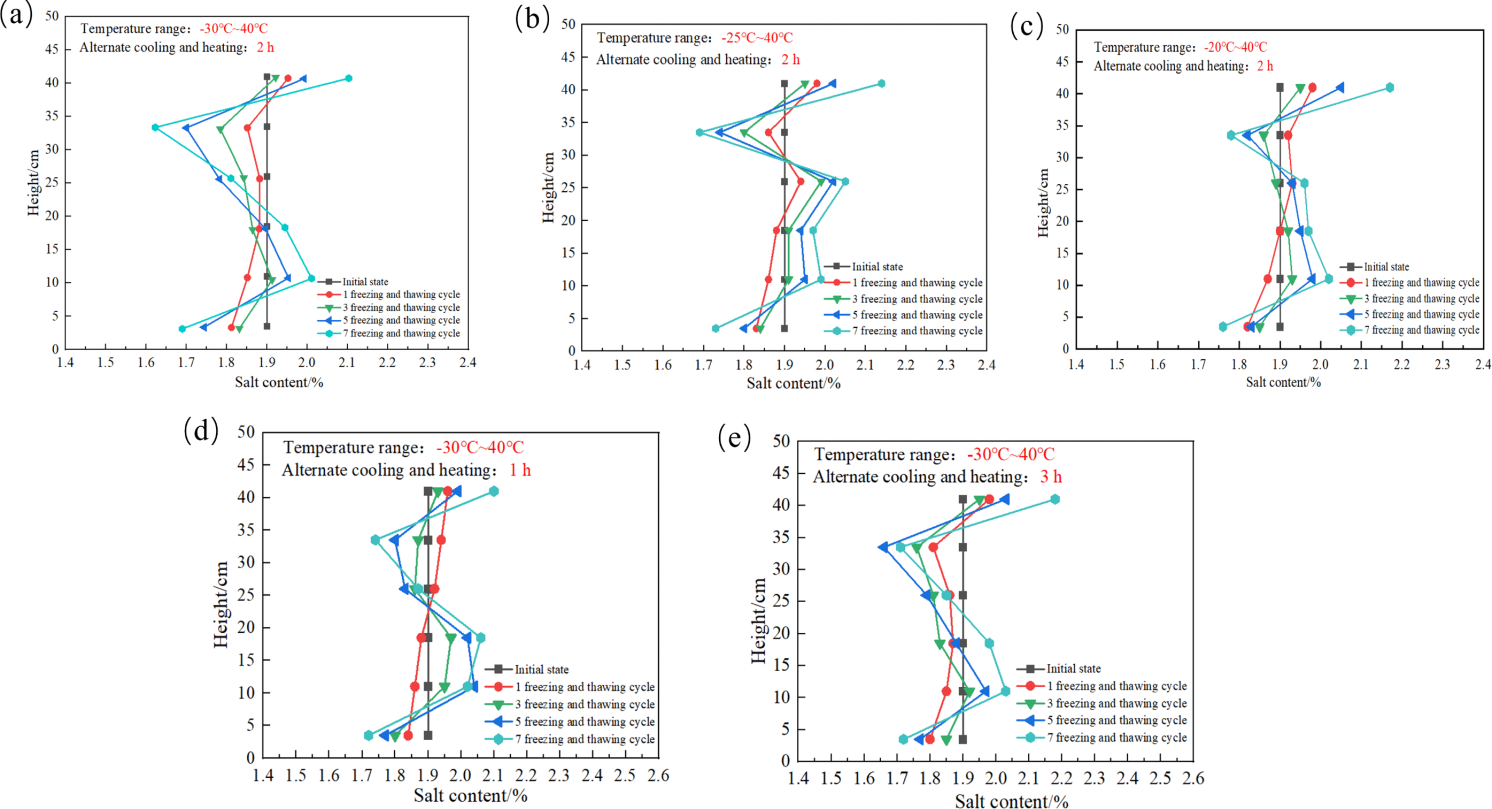
Salt distribution inside the soil column: (a) SC-A1, (b) SC-A2, (c) SC-A3, (d) SC-A4, (e) SC-A5.

The distribution pattern of salts within the SC is shown in Fig 11, which shows that the salinity in SC follows an “S” pattern in the direction of the height of the SC, and after 7 FTCs, the salt content at the SC’s upper level increases, whereas the salt content at the lower level decrease. The above phenomenon is manifested by the fact that the salts show accumulation at the top of the SC and dissipation at the bottom of the column. However, there is no significant correspondence between salt and water migration.

Comparison of Fig 11(a)–(c) shows that during the initial FTC, the temperature at the cold end decreases, resulting in salt accumulation and dissipation at varying depths, and there are differences in the salt distributions between the A2 and A3 SCs at 33.5 cm. This occurs because the freezing of the SC is weakened by the increase in temperature at the cold end. As a result, when the temperature at the cold end is −20°C, less salt migrates at the top of the SC, whereas more salt migrates in the middle of the SC under the action of the soil‒water potential, resulting in the accumulation of salt at 33.5 cm. With increasing number of FTCs, the accumulation and dissipation of salt increase; that is, the number of freeze‒thaw cycles increases the degree of soil salinization. With increasing numbers of FTCs, the interior of the SC gradually loosens, and the impact of the temperature gradient and soil‒water potential on soil salt migration gradually increases, which results in the gradual accumulation of salt at the top and in the lower parts of the SC. When the number of FTCs reaches 7, the salt content at the top of the SC gradually increased with the increase of the cold end temperature, which was opposite to the change rule of the water content at the top of the SC. When the cold end temperature was −30°C, the salt content showed the phenomenon of salt dissipation at the 26.0 cm measuring point, and as the cold end temperature increased, the salt content showed the phenomenon of salt accumulation at the 26.0 cm measuring point. The above phenomenon shows that the increase in cold end temperature increases the location of salt accumulation inside the SC.

Comparison of Fig 11 (a), (d) and (e) shows that the salt content at the top of the SC is 2.09%, 2.11% and 2.18% when alternating cooling and heating times is 1 hour, 2 hours and 3 hours, respectively. The above phenomenon shows that the salt content at the top of the SC increases gradually with the increase of alternating cooling and heating times. This is due to the fact that the longer alternating cooling and heating times, the greater the water migration, and at this time, the greater the amount of salt with water migration. In addition, with the increase in the number of FTCs, the salts accumulated inside the SC at the 11.0 cm, 18.5 cm and 41.0 c measurement points, while they dissipated inside the SC at the 3.5 cm, 26.0 cm and 33.5 cm measurement points. The above phenomena illustrate that the FTC aggravates salinization in some parts of the SC.

### 4.5. Pattern of soil deformation

**Fig 12:**
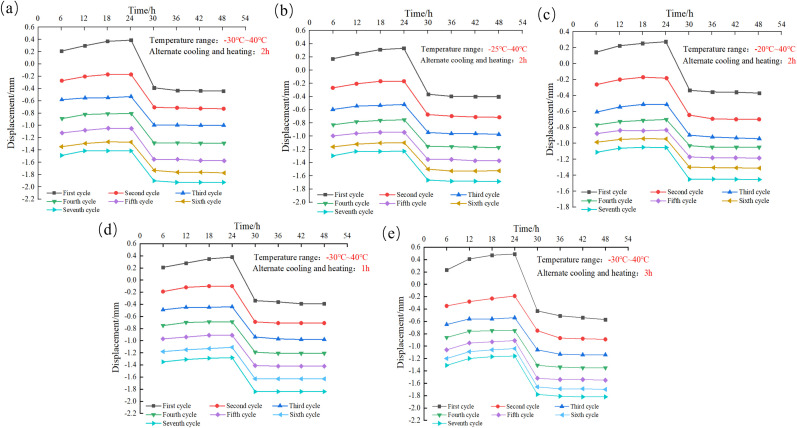
Displacement of SC samples under the action of large temperature differences: (a) SC-A1, (b) SC-A2, (c) SC-A3, (d) SC-A4, (e) SC-A5.

Preliminary studies on the subject have indicated that the deformation of clayey sand during the FTC is primarily attributable to frost and settlement deformations, resulting in a significant amount of settlement [33]. As shown in Fig 12, in this test, the displacement of the SCs increased with the increase of the number of FTCs, which showed the cumulative nature of solution subsidence.

Sodium sulfate demonstrates sensitivity to temperature changes. As a result, its solubility decreases rapidly during cooling, leading to the attainment of a supersaturated state within the sodium sulfate solution, thus absorbing water and crystallizing into sodium sulfate decahydrate. Part of the crystalline salt fills the pores of the soil particles, whereas the other part changes the position between the soil particles and breaks the soil structure, causing the soil to swell. As the temperature continues to decrease, the number of sodium sulfate decahydrate crystals that precipitate from the sodium sulfate solution in the soil also increases. When the internal temperature of the soil decreases to below the freezing point, the water in the soil begins to freeze, and ice crystals are produced, resulting in frost heave of the soil. Therefore, during the freezing process, the volume of a sample constantly increases. During the melting process, when the internal temperature of the soil increases to above the freezing point, the ice crystals in the soil begin to melt. At this time, the soil particles begin to fall back due to the loss of ice crystal support, thus reducing the frost-heave amount of the soil. As the temperature continues to rise, the solubility of sodium sulfate increases rapidly, and sodium sulfate crystals begin to dissolve. At this time, the soil particles begin to fall back due to the loss of support from the sodium sulfate crystals. As a result, the degree of soil salt expansion decreases. Under the action of the FTC, the water, salt and soil particles in the soil are redistributed, the soil becomes loose, the porosity increases, and settlement occurs under the action of self-gravity. As the number of FTCs increases, the amount of salt swelling and frost-heave decreases gradually, and the number of particles falling back is greater than the volume of salt swelling. During the next FTC, the number of particles falling back continues to be greater than the volume of salt swelling, indicating the increasing risk of collapsibility.

Comparing Fig 12(a)–(c), it can be seen that the displacements produced by SCs A1-A3 at the end of the 1st FTC are −0.46 mm, −0.39 mm, and −0.37 mm, respectively, and the final displacements produced by SCs A1-A3 at the end of the 7th FTC are −1.92 mm, −1.68 mm, and −1.44 mm, and the displacements of the SCs are negative, which means that the deformation of the SCs are settlement deformation. Based on this, as the cold end temperature increases, the settlement of the SC decreases. As the number of FTCs increases, the settlement of the SC increases. When the number of FTCs reaches 4 times, the settlement of SC-A3 gradually tends to stabilize; when the number of FTCs reaches 6 times, the settlement of SCs A1 and A2 gradually tends to stabilize. When the cold end temperature increases, the freezing front surface increases, resulting in the depth of the frozen zone of the soil body decreases, and the soil particles, moisture and salt inside the soil body are redistributed in a small range when the FTC is carried out, resulting in small structural changes of the soil body, which is ultimately manifested in the small amount of settlement of the soil body of A3, and the amount of settlement of the SC of A3 begins to tend to be stabilized after experiencing a relatively small number of FTCs.

Comparison of Fig 12 (a) (d) and (e) shows that the increase in the alternating cooling and heating times did not significantly affect the final deformation of the SC at the end of the seventh FTC. It should be pointed out that the deformation of the SC gradually stabilizes after the number of FTCs reaches 6 when alternating cooling and heating times is 2 and 3 hours, and the deformation of the SC does not stabilize with the increase of the number of FTCs when the alternating cooling and heating times is 1 hour.

## 5. Discussion

It can be seen from the results of the above tests that after 7 FTCs, the water content of each measuring point inside the SC was higher than the initial value; the distribution of water and salt inside the SC was not the same. However, Sarsembayeva et al. found that the water content in the middle of the SC was lower than the initial water content through the indoor WSM test; the distribution of water and salt inside the SC was the same [34]. The methods of the two tests mentioned above were similar, but the test results were different.

WSM under large temperature differences can be regarded as the processes of freezing at hypothermia and evaporation at elevated temperature. In the freezing stage, the unfrozen moisture content at the surface of the SC decreases, at this time, the soil water potential at the base of the SC is high and the soil water potential at the top is low, and the water continuously migrates to the top of the SC under the action of the soil water potential. Most scholars have verified the above viewpoints through experiments [16,20].

Zhou used 15°C at the low-temperature and 35°C at the high-temperature to conduct numerical simulation and indoor WSM test on salty soil, pointing out that the water will migrate to the low-temperature end in the form of vapor; the salt will be gathered at the high-temperature end due to desorption, which will result in the phenomenon that there are more salts at the high-temperature end and fewer salts at the low-temperature end [35].

Based on this theory, when the SC is in the melting stage, the temperature at the top of the SC is high, the water vapor pressure and the desorption of salts become stronger, and then the water will be dispersed to the low-temperature end in the form of vapor, and the salts will be gathered at the high-temperature end. In the middle of the SC, the temperature is low and the water vapor pressure is small, at this time, the water migrates to the low-temperature end in the form of liquid water, and the salts also migrate in the form of water, resulting in the accumulation of salts in the middle of the SC.

From the above explanation, it can be seen that during the melting stage, moisture migration in the form of vapor molecules diffuses to the low-temperature end, and eventually condenses into liquid water in the middle of the SC, resulting in a higher water content in the middle of the SC than the initial value. In the test used by Sarsembayeva et al. moisture migrated continuously towards the top under the effect of soil water potential, causing the water content in the middle of the SC to be lower than the initial value.

As can be seen in Fig 11 (a), (b) and (c), the lower the temperature at the cold end, the lower the accumulation of salts at the top of the SC. This did not show the phenomenon that the lower the temperature at the cold end, the stronger the suction for salts. The generation of this phenomenon can be explained by the freezing front [32]. In this experiment, the alternation time between hot and cold was set to be 2 hours, and the lower cold end temperature would result in a faster cooling rate. Therefore, when the cold end temperature is −30°C, the faster the cooling rate inside the SC, the top of the SC will be preferentially reduced to the freezing point, i.e., freezing fronts will be preferentially generated at the top of the SC. The generation of freezing fronts prevents salt from migrating to the top of the SC, so the earlier the freezing fronts are generated, the less salt will migrate.

## 6. Conclusion

In this work, saline soils in the western region of Uzbekistan were modified, and the effects of different cold-end temperatures and alternating cooling and heating times on the WSM patterns under the effect of large temperature differences were obtained by indoor WSM tests. The main conclusions of this research are as follows:

(1) Under the action of a large temperature difference, the curve showing the temperature change inside the SC appears as a “wave”, the curve showing the water content appears as a “C”, and the curve showing the salt content appears as an “S”.
(2) Under the action of large temperature differences, Water transport and salt transport did not show a clear correspondence. During the freezing process, water migrates from the bottom up under the action of the soil‒water potential and temperature gradient, and salt migrates from the bottom up, with water as the carrier, showing the pattern of “salt traveling with water”. During the melting process, the water at the top of the SC moves and diffuses from top to bottom in the form of steam molecules, while salt collects at the high temperature end due to desorption.
(3) The deformation of remolded saline soil during the first FTC involves mainly salt and frost heaving and deformation due to settlement. With an increasing number of FTCs, the collapsibility of saline soil increases. At the end of the seventh FTC, the accumulation of collapsible saline soil increases with decreasing cold-end temperature, and the change in the alternating cooling and heating times did not significantly affect the amount of deformation of the saline soils.
(4) The longer the alternating cooling and heating times, the higher the moisture and salt content at the top of the soil column; the lower the temperature at the cold end, the lower the moisture content at the top of the soil column and the higher the salt content. In addition, an increase in the number of FTCs increases the degree of soil salinization.

## Supporting information

S1 File Supporting data. (ZIP)

S2 File Inclusivity-in-global-research-questionnaire. (DOCX)
